# The pathogenic intestinal spirochaete *Brachyspira pilosicoli* forms a diverse recombinant species demonstrating some local clustering of related strains and potential for zoonotic spread

**DOI:** 10.1186/1757-4749-5-24

**Published:** 2013-08-16

**Authors:** Eugene Neo, Tom La, Nyree Dale Phillips, Mohammad Yousef Alikani, David J Hampson

**Affiliations:** 1 School of Veterinary and Life Sciences, Murdoch University, Murdoch, 6150 Western Australia, Australia; 2 Faculty of Medicine, Hamadan University of Medical Sciences, Hamadan, Iran

**Keywords:** *Brachyspira pilosicoli*, Spirochaete, Recombination, MLST, Zoonosis

## Abstract

**Background:**

*Brachyspira pilosicoli* is an anaerobic spirochaete that can colonizes the large intestine of many host species. Infection is particularly problematic in pigs and adult poultry, causing colitis and diarrhea, but it is also known to result in clinical problems in human beings. Despite the economic importance of the spirochaete as an animal pathogen, and its potential as a zoonotic agent, it has not received extensive study.

**Methods:**

A multilocus sequence typing (MLST) method based on the scheme used for other *Brachyspira* species was applied to 131 *B*. *pilosicoli* isolates originating from different host species and geographical areas. A variety of phylogenetic trees were constructed and analyzed to help understand the data.

**Results:**

The isolates were highly diverse, with 127 sequence types and 123 amino acid types being identified. Large numbers (50-112) of alleles were present at each locus, with all loci being highly polymorphic. The results of Shimodaira-Hasegawa tests identified extensive genetic recombination, although the calculated standardized index of association value (0.1568; P <0.0005) suggested the existence of some clonality. Strains from different host species and geographical origins generally were widely distributed throughout the population, although in nine of the ten cases where small clusters of related isolates occurred these were from the same geographical areas or farms/communities, and from the same species of origin. An exception to the latter was a cluster of Australian isolates originating from pigs, chickens and a human being, suggesting the likelihood of relatively recent transmission of members of this clonal group between species.

**Conclusions:**

The strongly recombinant population structure of *B*. *pilosicoli* contrasts to the more highly clonal population structures of the related species *Brachyspira hyodysenteriae* and *Brachyspira intermedia*, both of which are specialized enteric pathogens of pigs and poultry. The genomic plasticity of *B*. *pilosicoli* may help to explain why it has been able to adapt to colonize the large intestines of a wider range of hosts compared to other *Brachyspira* species. The identification of a clonal group of isolates that had been recovered from different host species, including a human being, suggests that zoonotic transmission by *B*. *pilosicoli* may occur in nature. Evidence for local transmission between the same host species also was obtained.

## Introduction

The genus *Brachyspira* includes seven officially named and several unofficially named species of anaerobic spirochaetes that colonize the large intestine of mammals and birds [1]. The three most commonly reported pathogenic species are *Brachyspira hyodysenteriae*, the agent of swine dysentery, *Brachyspira intermedia*, a pathogen mainly of adult chickens, and *Brachyspira pilosicoli*, the cause of a condition that has been called ‘intestinal spirochaetosis’. *B*. *pilosicoli* has a broader host range than the other two main pathogenic species, colonizing various species of mammals and birds, as well as human beings [2,3].

Infections with *B*. *pilosicoli* are particularly common in intensively housed pigs and chickens, in which they cause depressed rates of growth and production. Colonization also commonly occurs in human beings living in crowded and unhygienic conditions in developing countries [4-7], as well as amongst homosexual males [8]. Individuals colonized with *B*. *pilosicoli* may develop focal colitis and chronic diarrhoea, with abdominal pain, failure to thrive and rectal bleeding. An *in vitro* study using Caco-2 cells has shown that *B*. *pilosicoli* strains initially target the cell junctions, where one cell end of the spirochaete invaginates into the Caco-2 cell membranes [9]. The whole cell surface progressively becomes colonized by attached spirochaetes, forming a “false brush border”. In this model colonized monolayers demonstrated accumulation of actin at the cell junctions, loss of tight junction integrity, condensation and fragmentation of nuclear material consistent with apoptosis, and a significant up-regulation of interleukin-1beta and interleukin-8 expression. Besides colitis, a spirochaetaemia with *B*. *pilosicoli* has been recorded in immunocompromised or debilitated human beings [10,11], and systemic spread involving the liver also has been described in experimentally infected chickens [12].

*B*. *pilosicoli* may be found in water contaminated with faeces and on foodstuffs, and hence has potential importance as a water-borne or food-borne zoonotic pathogen [5,13,14].

Earlier studies using multilocus enzyme electrophoresis (MLEE) have suggested that *B*. *pilosicoli* is a recombinant species [2], and that cross-species transmission is likely to occur [3]. A similar MLEE study with the related *B*. *hyodysenteriae* also suggested that this species is recombinant with an epidemic population structure [15]; however, more recent studies using multilocus sequence typing (MLST) have indicated that *B*. *hyodysenteriae* has a clonal population structure [16,17], as does *B*. *intermedia*[18]. These results now have left some uncertainty about the likely population structure of *B*. *pilosicoli*.

In an earlier genus-wide MLST study of *Brachyspira* species, 12 strains of *B*. *pilosicoli* were included in the analysis [19]; however, this was too few to deduce the population structure, and there have been no subsequent reports where MLST has been used to analyze *B*. *pilosicoli* isolates. Consequently the overall aim of the current study was to apply the previously developed but incomplete brachyspira MLST system to a large and diverse collection of *B*. *pilosicoli* strains to improve understanding of diversity, population structure, host-specificity and geographical links between strains.

## Results and discussion

In this study 131 *B*. *pilosicoli* strains and isolates from various countries and animal species that had been collected over three decades were used in an MLST scheme for *B*. *pilosicoli*. Between 50-112 alleles were identified at the seven MLST loci tested, and a total of 127 sequence type (ST) profiles were obtained (ST01 to ST127). These results demonstrated that high rates of genetic variation occur within the population. The data are summarized in Table 1, with allelic profiles for individual strains shown in Additional file 1: Table S1. The raw sequence data were deposited at the PubMLST site (http://pubmlst.org/brachyspira/). After the translation of nucleotides into amino acids, 16-72 alleles were identified at the various loci and 123 amino acid type (AAT) profiles were present (Table 1).

**Table 1:** Number of alleles, genetic diversity, GC content, and variable sites at the seven loci tested

**Loci**	**No. of alleles**	**h value**	**Sequence length**	**No. of variable sites**	**Variable sites%**	**% G + C content**	**Ln Likelihood**	**No. of amino acids**
*adh*	50	0.913	347	114	32.9	41.5	-1833.41711	24
*alp*	90	0.989	648	294	45.4	34.4	-5659.75171	67
*est*	95	0.989	498	419	84.1	34.2	-8054.48965	68
*gdh*	64	0.983	412	56	13.6	34.3	-1839.28238	16
*glp*	77	0.988	686	170	24.8	32.8	-3783.80503	38
*pgm*	112	0.986	743	377	50.7	33.1	-5930.31493	72
*thi*	90	0.992	745	630	84.6	39.2	-11915.65273	71
Mean h value		0.977						

The mean genetic diversity (h value) was 0.977, with diversity at the individual loci varying from 0.913 to 0.989 (Table 1). The extensive diversity that was identified in the population agreed with the results of the earlier MLEE study on *B*. *pilosicoli*[2]. Multilocus variable number tandem repeat analysis of *B*. *pilosicoli* also has shown considerable diversity, but the frequent occurrence of null alleles limits the use of the technique for detailed analysis of relationships between isolates [20].

The results of the Shimodaira-Hasegawa (SH) test for the seven loci are recorded in Additional file 2: Table S2, and they indicate that each tree had the best topology to explain the genetic relationship of the loci tested. The 35 concatenated trees constructed by using different combinations of three alleles were distinctively different from each another. Results for the four trees that showed the greatest difference are presented in Additional file 3: Table S3. These SH tests indicated that there is substantial recombination in the evolutionary history of *B*. *pilosicoli*, and that each gene analyzed was independently evolving. Thus, for each gene there was a significant difference in the Δ - ln L values of each tree and, furthermore, for each of the seven genes the maximum likelihood (ML) trees were no more similar in likelihood than the 200 random trees for each data set. Hence significant phylogenetic incongruence was revealed, implying that frequent recombination has obscured phylogenetic signals expected from direct inheritance of genes in the population.

**Figure 1:**
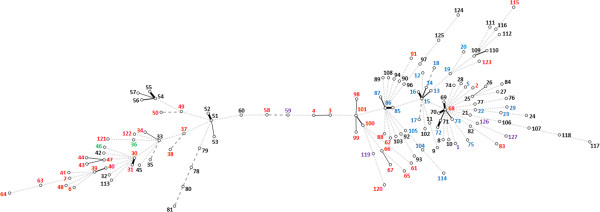
**Minimum spanning tree showing the MLST profiles of 131 *Brachyspira pilosicoli *strains with the host species of origin marked.** Each node corresponds to a sequence type (ST). The lines between STs show inferred phylogenetic relationships and are represented by bold, continuous, continuous thin, dashed and dotted lines according to the number of allelic mismatches between profiles (1, 2, 3, 4 and 5 or more, respectively). Host species of origin are indicated with coloured text (human (red circle), pig (black circle), chicken (blue circle), dog (violet circle), horse (green circle)).

**Figure 2:**
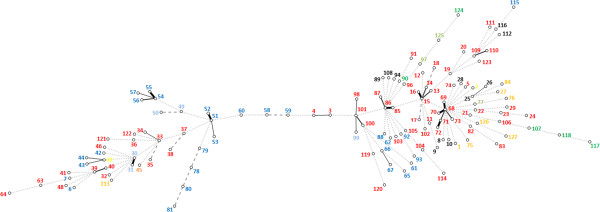
**Minimum spanning tree showing the origin of the *B*. *pilosicoli *strains.** The country of isolation is shown in coloured text (Australia (red circle), Sweden (black circle), Papua New Guinea (blue circle), USA (violet circle), Canada (green circle), Italy (light blue circle), France (yellow circle), UK (olive green circle), New Zealand (orange circle)).

The standardized index of association (I^S^_A_) value was calculated as 0.1568 (P < 0.0005), with a small but significant linkage disequilibrium being present in the population. The values are listed in Table 2. Despite the evidence for the population being recombinant, the value suggested that there was a limited degree of clonality within the species that was not masked by high rates of recombination. Consistent with this, all trees that were constructed showed deep branching but with a few small clusters of related isolates (see Additional file 4: Figure S1 as an example). Clustering could be most easily seen in a minimum spanning (MS) tree, which also is marked in colour to show the species of origin (Figure 1) and geographical origin (Figure 2).

**Table 2:** Index of association values generated in the START2 program

**Index of association**	**V**_**o**_	**V**_**e**_	**I**_**A**_	**I**^**S**^_**A**_	**Mean trial variance**	**Max trial variance**	**5% critical value**
	0.2667	0.1366	0.9516	0.1568	0.1367	0.1459	0.1399

Abbreviations; *V*_*o*_ observed variance, *V*_*e*_ expected variance, *I*_*A*_ index of association, *I*^*S*^_*A*_ standardized index of association.

The MS tree was divided into two main but linked parts, with more strains and STs located on the right hand side than on the left. Generally isolates from the same species and geographical origins were distributed throughout the tree, although all the isolates from chickens were located on the right hand side. This distribution might have been influenced by the relatively restricted range of isolates available for analysis, and more robust results will be obtained when more *B*. *pilosicoli* strains from different hosts and geographic areas are added to the PubMLST database. There was a limited degree of clustering of isolates (nine clusters: defined as isolates with allelic differences at only one or two loci), and in all but one case where this occurred the clustered isolates were from the same species and from the same geographical origin or farm/community. Hence they are likely to have represented a clonal group that has been transmitted locally. The exception was the largest cluster around ST68 that consisted of isolates from dispersed geographical locations in Australia, and from different host species. This cluster consisted of a isolate from a human child in the Kimberley region in the north of Western Australia, an isolate from a pig in Victoria, two isolates from pigs in the same piggery in the southwest of Western Australia, and two isolates from chickens in Queensland. The occurrence of isolates from one cluster in different species does suggest the possibility of recent cross-species transmission, although it is unlikely to have occurred recently in this case due to the wide geographical distances between the sites where the isolates originated. Possible mechanisms would be transmission through migratory bird species, or mechanical transmission associated with human activities.

By contrast to *B*. *pilosicoli*, when using MLST the species *B*. *hyodysenteriae* and *B*. *intermedia* both have been deduced to be essentially clonal [16,18]. Hence these three important pathogenic species in the same genus have different population structures. One interpretation could be that the latter two species have evolved relatively recently from single stable strains or clones that were derived from a highly recombinant ancestral species such as *B*. *pilosicoli*, and which have been successful in finding suitable specialized niches in specific host species. Another possibility could be that the recombinant *B*. *pilosicoli* developed from a more stable clonal ancestor following development or acquisition of more effective means for gene transfer and recombination.

**Figure 3:**
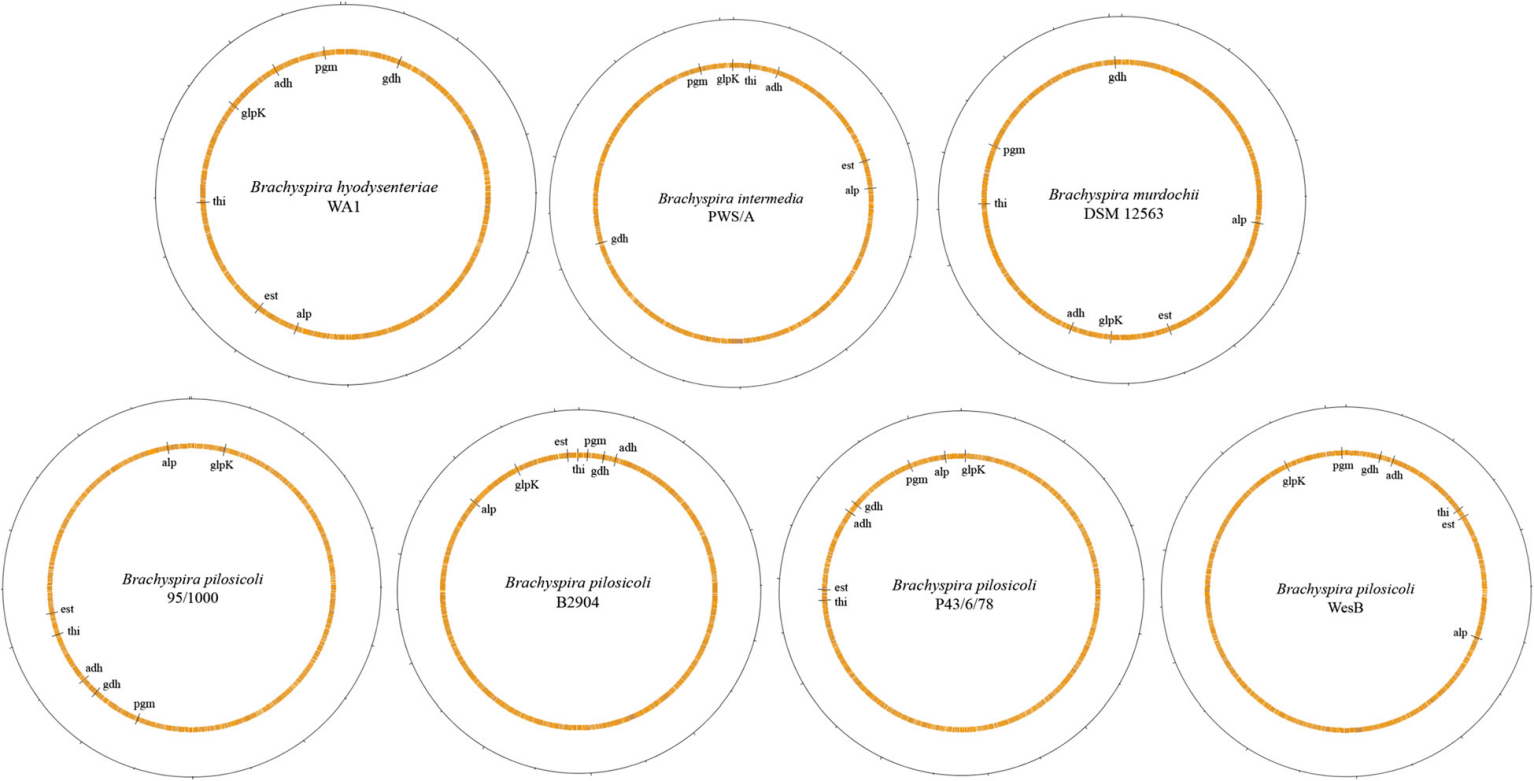
**Genome maps of *B*. *hyodysenteriae *WA1, *B*. *intermedia *PWS/A, *B*. *murdochii *DSM 12563 and the four publically available complete *Brachyspira pilosicoli *genomes (95/1000; B2904; P43/6/78**^**T**^**; WesB) showing the relative positions of the seven genes targeted for MLST.**

The source of the variation amongst *Brachyspira* species and strains is of considerable interest. Based on the high degree of conservation in the 16S rDNA sequences of the *Brachyspira* species it has been suggested that they have evolved relatively recently [1]. The location of the seven loci used in the MLST scheme mapped on seven complete *Brachyspira* genomes is shown as Figure 3. Not only do the relative positions of the loci vary greatly between species, but there are also remarkable differences between the locations in the four sequenced *B*. *pilosicoli* strains. These extensive genomic rearrangements within and across species demonstrate the plasticity of *Brachyspira* genomes.

One potential means for recombination may be the activity of bacteriophage-like gene transfer agents (GTA) that have been detected in various *Brachyspira* species, and which have the potential to facilitate gene transduction within or possibly even across species [21-23]. In addition, in the case of *B*. *pilosicoli*, recent analysis of the genomes of three sequenced strains identified genome rearrangements that largely correlated with the positions of mobile genetic elements [24]. Novel bacteriophages also were detected in the newly sequenced genomes, and clearly such genetic elements may have the potential to transduce genetic information and contribute to the recombination that has been recorded here. Interestingly the sizes of the genomes of three sequenced *B*. *pilosicoli* strains (B2904, WesB and 95/1000) were ~2,765, 2.890 and 2.596 Mb, respectively [24], while the genome of strain P43/6/78^T^ has been recorded as 2.56 Mb [25]. This variation in genome size with accompanying loss or gain of genes provides clear evidence for the genomic plasticity of *B*. *pilosicoli*.

## Conclusions

This study has confirmed that *B*. *pilosicoli* has a strongly recombinant population structure that contrasts to the more highly clonal population structures of the related pathogenic species *B*. *hyodysenteriae* and *B*. *intermedia*. *Brachyspira* species showed evidence of extensive rearrangement of MLST loci on their genomes, including across four previously sequenced *B*. *pilosicoli* strains. The greater genomic plasticity of the recombinant *B*. *pilosicoli* may help to explain why it can colonize the large intestines of a wider range of hosts compared to other *Brachyspira* species. The MLST system that was used was sufficiently sensitive to be able to detect a number of instances where closely related strains (clones) of *B*. *pilosicoli* were present in individual animals or people from the same farms or communities, as well as providing evidence for the potential for cross-species and zoonotic transmission by related *B*. *pilosicoli* strains.

## Methods

### *Brachyspira pilosicoli* strains and isolates

A total of 119 well-characterized strains and isolates of *B*. *pilosicoli* were obtained as frozen stock from the culture collection at the Reference Centre for Intestinal Spirochaetes at Murdoch University. They originated from different States of Australia (n = 66), Papua New Guinea (n = 29), the United States of America (n = 8), Canada (n = 5), Italy (n = 5), the United Kingdom (n = 3), France (n = 2) and New Zealand (n = 1). Sequence data for 12 Scandinavian and European strains (AN4170/01, AN991/02, AN76/92, AN497/93, C62, AN984/03, AN1085/02, AN652/02, AN2248/02, AN738/02, AN953/02 and C162) that were previously used in a *Brachyspira* genus-wide MLST study [19] were obtained from the PubMLST website (http://pubmlst.org/) and were included in this analysis.

The full collection, representing 131 isolates, came from a range of species, and consisted of 58 from pigs, 44 from human beings, 24 from chickens, five from dogs and two from horses. The names and origins of the isolates are listed in Additional file 1: Table S1. The identity of the isolates was confirmed using a species-specific PCR for *B*. *pilosicoli* incorporating 16S rDNA primers [26].

### Spirochaete culture and DNA extraction

The spirochaetes were propagated at 37°C for 5 days in Kunkle’s pre-reduced anaerobic broth containing 2% foetal bovine serum and a 1% ethanolic cholesterol solution [27]. Cells were harvested from culture by centrifuging at 10,000 g, and counted in a haemocytometer chamber under a phase contrast microscope at 40 times magnification.

For each strain, 10 ml of Trypticase Soy broth containing ~10^8^ cells/ml of *B*. *pilosicoli* was centrifuged at 5000 g. The supernatant was discarded and the pellet resuspended in an equal volume of phosphate buffered saline (pH 7.4) and heated at 95°C for 15 min to release the DNA, before storing at -20°C. The solution containing the extracted DNA was used as the template for the PCR reactions.

### Multilocus sequence typing (MLST)

The seven loci used in MLST were the same as those previously described for use with members of the genus *Brachyspira*[19]. These were the genes encoding for the conserved “housekeeping” genes alcohol dehydrogenase (*adh*), alkaline phosphatase (*alp*), esterase (*est*), glutamate dehydrogenase (*gdh*), glycerol kinase (*glpK*), phosphoglucomutase (*pgm*), and acetyl-CoA acetyltransferase (*thi*). The PCR primers used were the same as those used previously [16]. To confirm the conservation of these loci their positions were plotted on genomes of the available single strains of *B*. *hyodysenteriae* (WA1), *B*. *intermedia* (PWS/A^T^), *B*. *murdochii* (DSM12563) and four strains of *B*. *pilosicoli* (95/1000; B2904; WesB; P43/6/78^T^) [24,28-30].

PCR was performed on DNA from all the *B*. *pilosicoli* isolates, using 0.2 μl *Taq* DNA polymerase, 5 μl of 10× PCR buffers, 3 μl of 25 mM MgCl_2_, 5 μl of 8 mM dNTP, 5 μl of the forward and reverse primers, 12 μl of cresol red solution and 2 μl of template, with the reaction mix topped up with PCR-grade water to a final 50 μl volume. Each PCR reaction set included DNA from *B*. *pilosicoli* strain 95/1000 as a positive control and distilled water as a negative control. The PCR conditions were 95°C for 2 min, followed by 33 cycles at 95°C for 30 sec, 50°C for 15 sec, 72°C for 1 min for every 1 kbp of product, and a final extension period of 5 min at 72°C before holding at 14°C.

The PCR products were subjected to electrophoresis in a 1% agarose gel in a Bio-Rad Sub-Cell® GT Agarose Gel electrophoresis unit at 120 V for 30 min. A 1 Kbp ladder marker was added to the first and last well of each row to allow estimates of the molecular masses of the samples. The gel was stained by immersion in an ethidium bromide solution at a concentration of 0.5 μg/ml for 30 mins, and the DNA was visualized over a UV illuminator (Biorad Chem Doc XRS Universal Hood).

For sequencing, the PCR products were purified with the Wizard® SV Gel and PCR Clean-Up System Kit (Promega) following the manufacturer’s instructions, then PCR was performed on the purified products using the BigDye Terminator v3.1 Cycle Sequencing Kit (Applied Biosystems, Foster City, USA) in a 96 well plate, according to the manufacturer’s instructions, using 10 μl of a single primer instead of 5 μl of both forward and reverse primer in each reaction. The amplified products were purified using ethanol precipitation and the pellet was held at 4°C before being sequenced with the ABI 373A sequencing system (Applied Biosystems).

### Analysis

The sequences were analyzed and assembled using the Bioedit Sequence Alignment Editor [31]. The sequences for each locus were aligned using the ClustalW program (EMBL-EBI, European Bioinformatics Institute [http://www.ebi.ac.uk/Tools/msa/clustalw2/]) and *B*. *pilosicoli* strain 95/1000 sequence as the standard for the process.

Two methods were used to generate phylogenetic trees. In the first the aligned sequences for each of the seven loci were analyzed using the non-redundant databases (NRDB) program (http://pubmlst.org/analysis/) to identify strain sequences that were identical to each other. Each unique nucleotide sequence was then assigned with a different allele number. The allelic profile for each isolate was determined and consisted of a line listing the allele number for each locus in turn. Isolates were assigned a sequence type (ST) according to their allelic profiles. Isolates were considered genetically identical and belonging to the same ST if their sequences were identical at all seven loci.

The allelic profile was then entered into the dataset of the START2 program and rooted phylogenetic trees (“consensus trees”) with 1000 bootstrap replicates were generated from the data matrix using the ‘Unweighted Pair Group Method with Arithmetic Mean’ (UPGMA) and ‘Neighbour-Joining’ (NJ) method with the ‘Maximum likelihood’ (ML) models [32]. A minimum spanning tree also was generated using the Bionumerics Software (version 7.1, Applied Maths), and colour coded according to the species of origin and geographical origin of the isolates.

The allelic profile was used to calculate genetic diversity, as previously described [33]. To help determine whether recombination had occurred within the *B*. *pilosicoli* population, the START2 program was used to estimate the degree of linkage disequilibrium in the population by calculating the index of association (I_A_) and the standardized index of association (I^S^_A_) [34].

The second method of generating phylogenetic trees was by concatenating the nucleotide sequences for the seven genes of each isolates in the order *adh*, *pgm*, *est*, *glp*, *gdh*, *thi* and *alp* (the same order previously used for other *Brachyspira* species).

All sequences were placed in a single FASTA formatted file and aligned with ClustalW before being converted to the MEGA format (http://ccg.murdoch.edu.au/tools/clustalw2mega/). UPGMA and NJ trees were constructed from the aligned DNA sequences using the MEGA v4.0.2 program [35].

To verify the topology of the phylogenetic trees, the Shimodaira-Hasegawa (SH) test was carried out using the Phylip v3.69 program [36] to detect significant differences amongst the trees extrapolated for each gene. This analysis was carried out by estimating the maximum likelihood (ML) trees for each of the seven genes, and then comparing, in turn, the difference in log likelihood (Δ - ln L) between each of the seven topologies on each of the seven genes. Randomization tests were used to assess the extent of congruence amongst the seven ML gene trees, and the Δ - ln L values for each of the seven ML trees fitted to each of the seven genes were compared to the equivalent values computed for 200 random trees created from each gene.

To test whether the genetic variation at different loci were independent of one another, 35 ML trees were constructed from concatenated sequences of sets of three random loci for the 127 STs. It was expected that if there were associations between the loci, there would be little variation between the different trees.

## Competing interests

All authors declare that they have no conflicts of interests.

## Authors’ contributions

EN, TL and DJH designed the experiments and they were performed by EN, NDP, TL and MYA. EN, TL and DJH analysed the data and DJH and EN wrote the manuscript. All authors approved the manuscript for publication.

## Supplementary Material

Additional file 1: Table S1 The names of the 131 isolates, species from which they were isolated, the country of isolation, the sequence type (ST) to which they were assigned in the study and the allelic number assigned to the seven loci. The shaded boxes represent nine sets of isolates in different adjacent STs that differ at only one or two loci, and were each defined as a cluster. Click here for file

Additional file 2: Table S2 Results of the Shimodaira-Hasegawa test for the seven loci. Click here for file

Additional file 3: Table S3 Results of the Shimodaira-Hasegawa test on the four concatenated trees that showed the greatest difference with combinations of three loci. Click here for file

Additional file 4: Figure S1 Neighbour joining tree using the consensus sequences of the 131 *B. pilosicoli* isolates. A few localized clusters of isolates can be seen, with the largest being ST68 – ST73. Click here for file
