# Correction to: The pathogenic intestinal spirochaete *Brachyspira pilosicoli* forms a diverse recombinant species demonstrating some local clustering of related strains and potential for zoonotic spread

**DOI:** 10.1186/s13099-019-0328-3

**Published:** 2019-10-14

**Authors:** Eugene Neo, Tom La, Nyree Dale Phillips, Mohammad Yousef Alikani, David J. Hampson

**Affiliations:** 1 School of Veterinary and Life Sciences, Murdoch University, Murdoch, WA 6150 Australia; 2 Faculty of Medicine, Hamadan University of Medical Sciences, Hamadan, Iran

## Correction to: Gut Pathogens 5:24 (2013) 10.1186/1757-4749-5-24

The article published in 2013 [1] described the use of the available *Brachyspira* multilocus sequence typing (MLST) scheme [2] to characterize the population structure of the intestinal spirochete *Brachyspira pilosicoli*. It used sequences of seven loci that were amplified from 131 strains that had been isolated from different geographical origins and species.

Recently we sequenced the full genomes of 34 of these isolates, and identified some sequence errors for the genes used in the MLST analysis. We attempted to grow the remaining 97 isolates, but 17 could not be recovered from storage. The sequences at the seven loci for the remaining 80 isolates were determined using the Dye Termination method, and additional minor sequence errors were identified. All these sequencing errors have been corrected in the PubMLST site (http://pubmlst.org/brachyspira/). Table 1 in the original article has been updated in this correction, and Figs. 1 and 2 have been redrawn. The main conclusions of the original work have not been changed. The isolates are highly diverse, with 94 sequence types. Large numbers of alleles were found at each locus (36 to 74). The calculated index of association value (1.196; P > 0.001) suggests some clonality. Corrected Table 1 and Figs. 1 and 2 are given here.

**Table 1:** Number of alleles, genetic diversity, GC content, and variable sites at the seven loci tested

Loci	No. of alleles	h value	Sequence length	No. of variable sites	Variable sites %	%G + C content	No. of amino acids
*adh*	36	0.906	347	111	32	41.6	13
*alp*	74	0.989	641	186	29	34.2	47
*est*	64	0.985	487	355	72.9	33.8	37
*gdh*	49	0.975	412	50	12.1	34.2	13
*glp*	54	0.984	686	131	19	32.8	16
*pgm*	72	0.991	743	164	22.1	33	29
*thi*	68	0.989	745	442	59.3	39.1	48
Mean h value		0.974

**Fig. 1:**
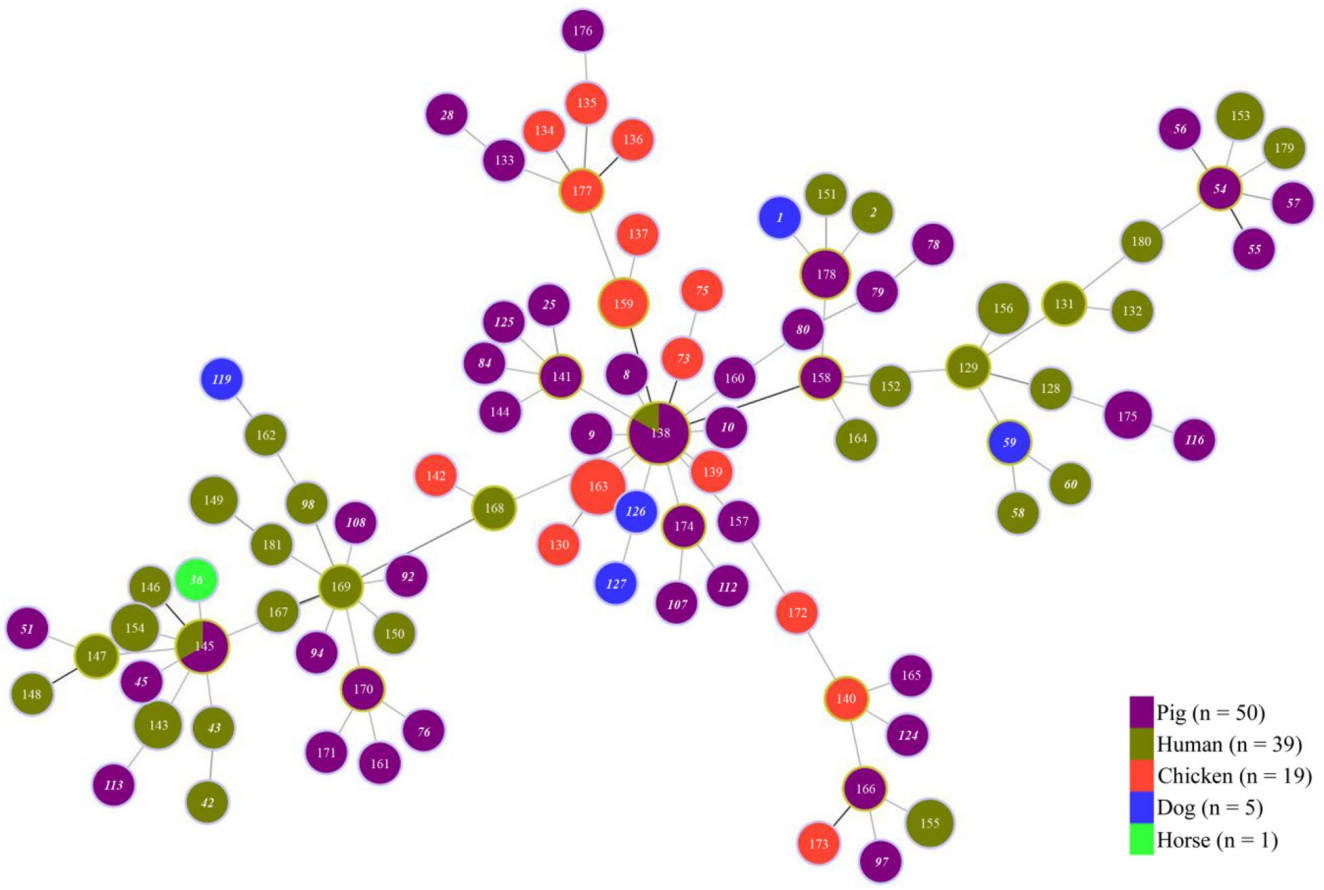
Minimum spanning tree showing the MLST profiles of 114 *Brachyspira pilosicoli* strains with the host species of origin marked. Each node corresponds to a sequence type (ST). The lines between STs show inferred phylogenetic relationships and are represented by bold, continuous and continuous thin lines according to the number of allelic mismatches between profiles. Host species of origin are indicated with coloured circles as indicated in the legend

**Fig. 2:**
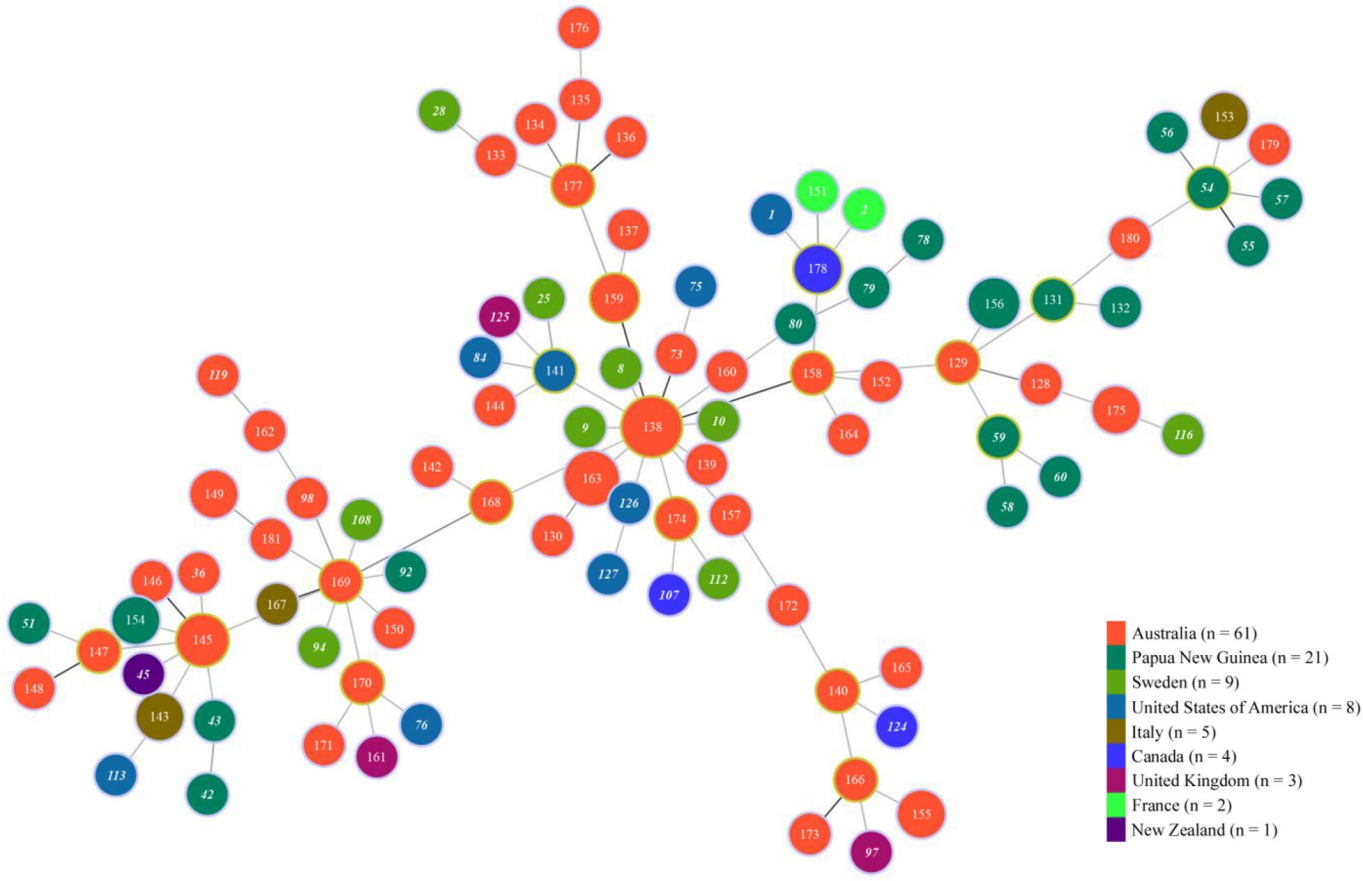
Minimum spanning tree showing the origin of the *B*. *pilosicoli* strains. Each node corresponds to a sequence type (ST). The lines between STs show inferred phylogenetic relationships and are represented by bold, continuous and continuous thin lines according to the number of allelic mismatches between profiles. The country of isolation is shown by coloured circle as indicated in the legend
