# Supplementary material for: The pathogenic intestinal spirochaete Brachyspira pilosicoli forms a diverse recombinant species demonstrating some local clustering of related strains and potential for zoonotic spread
Source: Gut Pathog. 2013 Aug 16;5:24. doi: 10.1186/1757-4749-5-24 (PMC3751851; doi:10.1186/1757-4749-5-24)
Supplement: Additional file 1: Table S1 — The names of the 131 isolates, species from which they were isolated, the country of isolation, the sequence type (ST) to which they were assigned in the study and the allelic number assigned to the seven loci. The shaded boxes represent nine sets of isolates in different adjacent STs that differ at only one or two loci, and were each defined as a cluster. [file 1757-4749-5-24-S1.docx]

**Supplementary Table 1**. The names of the 131 isolates, species from which they were isolated, the country of isolation, the sequence type (ST) to which they were assigned in the study and the allelic number assigned to the seven loci. The shaded boxes represent nine sets of isolates in different adjacent STs that differ at only one or two loci, and were each defined as a cluster.

| Isolate name | Species of origin | Country of isolation* | ST | *adh* | *alp* | *est* | *gdh* | *glp* | *pgm* | *thi* |
| --- | --- | --- | --- | --- | --- | --- | --- | --- | --- | --- |
| 24072-93A | Dog | USA | 1 | 1 | 1 | 1 | 1 | 1 | 1 | 1 |
| 28/94 | Human | France | 2 | 2 | 2 | 2 | 2 | 2 | 2 | 2 |
| Tomara | Human | Australia | 3 | 3 | 3 | 3 | 3 | 3 | 3 | 3 |
| Quentin F1 | Human | Australia | 4 | 4 | 3 | 4 | 3 | 3 | 4 | 3 |
| Q98.0028.3 | Chicken | Australia | 5 | 5 | 4 | 5 | 4 | 3 | 5 | 4 |
| V1 H11 | Human | PNG | 6 | 6 | 5 | 6 | 5 | 3 | 6 | 5 |
| V1 H141 | Human | PNG | 7 | 7 | 6 | 6 | 6 | 4 | 7 | 5 |
| AN652/02 | Pig | Sweden | 8 | 5 | 33 | 34 | 25 | 29 | 42 | 31 |
| AN984/03 | Pig | Sweden | 9 | 5 | 34 | 35 | 26 | 31 | 43 | 32 |
| AN1085/02 | Pig | Sweden | 9 | 5 | 34 | 35 | 26 | 30 | 43 | 32 |
| AN2448/02 | Pig | Sweden | 10 | 5 | 32 | 33 | 24 | 28 | 41 | 30 |
| L72 | Pig | Australia | 11 | 5 | 35 | 36 | 27 | 31 | 44 | 33 |
| Q97.000.6.10 | Chicken | Australia | 12 | 5 | 36 | 37 | 27 | 32 | 45 | 34 |
| Q98.0072.37 | Chicken | Australia | 13 | 5 | 37 | 38 | 27 | 18 | 46 | 35 |
| Q98.0026.12 | Chicken | Australia | 14 | 5 | 37 | 12 | 27 | 18 | 47 | 35 |
| Q98.0062.14 | Chicken | Australia | 15 | 5 | 37 | 39 | 27 | 18 | 15 | 35 |
| Q98.0027.36 | Chicken | Australia | 16 | 5 | 37 | 39 | 27 | 18 | 48 | 35 |
| Q97.2224.3.1 | Chicken | Australia | 17 | 5 | 38 | 40 | 27 | 18 | 49 | 36 |
| Q98.0026.11 | Chicken | Australia | 18 | 5 | 39 | 12 | 27 | 33 | 50 | 37 |
| Q96.1037.0 | Chicken | Australia | 19 | 5 | 40 | 30 | 28 | 34 | 46 | 38 |
| Q98.0228.5.7 | Chicken | Australia | 20 | 5 | 41 | 41 | 28 | 35 | 51 | 39 |
| Wand F | Pig | Australia | 21 | 5 | 49 | 48 | 34 | 43 | 59 | 24 |
| Q97.3008.4.2 | Chicken | Australia | 22 | 5 | 42 | 42 | 29 | 36 | 52 | 40 |
| Q98.000.6.1 | Chicken | Australia | 23 | 5 | 43 | 42 | 30 | 37 | 53 | 41 |
| OF 2 | Pig | Australia | 24 | 5 | 44 | 43 | 30 | 5 | 54 | 42 |
| C162 | Pig | Sweden | 25 | 5 | 45 | 44 | 31 | 38 | 55 | 16 |
| AN953/02 | Pig | Sweden | 26 | 16 | 45 | 44 | 31 | 39 | 55 | 99 |
| D9201243 | Pig | USA | 27 | 5 | 46 | 45 | 31 | 40 | 56 | 43 |
| AN738/02 | Pig | Sweden | 28 | 5 | 47 | 46 | 32 | 41 | 57 | 99 |
| Q94.0354.0.6 | Chicken | Australia | 29 | 41 | 80 | 42 | 57 | 71 | 102 | 81 |
| HRM 2B | Human | Italy | 30 | 9 | 53 | 7 | 38 | 45 | 63 | 47 |
| HRM 2A | Human | Italy | 31 | 9 | 53 | 7 | 38 | 45 | 64 | 47 |
| Q95.3281.0 | Pig | Australia | 32 | 20 | 54 | 51 | 38 | 46 | 65 | 48 |
| OF15 | Pig | Australia | 33 | 9 | 56 | 54 | 16 | 44 | 68 | 50 |
| H43-2 | Human | Australia | 34 | 9 | 56 | 55 | 16 | 44 | 69 | 51 |
| OF 11 | Pig | Australia | 35 | 9 | 56 | 56 | 16 | 48 | 70 | 52 |
| 2152 | Horse | Australia | 36 | 9 | 57 | 49 | 16 | 40 | 71 | 53 |
| Joyceline | Human | Australia | 37 | 9 | 58 | 57 | 40 | 44 | 72 | 42 |
| H21 | Human | Australia | 38 | 9 | 58 | 58 | 40 | 49 | 73 | 54 |
| H4-2 | Human | Australia | 39 | 8 | 7 | 6 | 7 | 5 | 8 | 5 |
| H38-2 | Human | Australia | 40 | 8 | 7 | 7 | 7 | 5 | 9 | 5 |
| Barney | Human | Australia | 41 | 8 | 8 | 8 | 7 | 6 | 10 | 6 |
| V1 H 12 | Human | PNG | 42 | 9 | 9 | 9 | 7 | 7 | 11 | 7 |
| V1 H 116 | Human | PNG | 43 | 9 | 3 | 9 | 7 | 7 | 12 | 8 |
| V1 H 103 | Human | PNG | 44 | 9 | 3 | 10 | 7 | 7 | 13 | 7 |
| NZ 91/31349 | Pig | NZ | 45 | 17 | 50 | 49 | 35 | 44 | 60 | 44 |
| Apr-52 | Horse | Australia | 46 | 18 | 51 | 49 | 36 | 45 | 61 | 45 |
| BR81/80 | Human | France | 47 | 19 | 52 | 50 | 37 | 45 | 62 | 46 |
| Gap418 | Human | Australia | 48 | 10 | 10 | 11 | 8 | 5 | 14 | 9 |
| HRM7 | Human | Italy | 49 | 25 | 62 | 62 | 12 | 3 | 78 | 58 |
| HRM7A | Human | Italy | 50 | 25 | 62 | 63 | 12 | 54 | 79 | 59 |
| GP 24 | Pig | PNG | 51 | 27 | 58 | 65 | 12 | 56 | 80 | 60 |
| GP 17 | Pig | PNG | 52 | 27 | 58 | 64 | 12 | 56 | 80 | 60 |
| GP 36 | Pig | PNG | 52 | 27 | 58 | 64 | 12 | 56 | 80 | 60 |
| GP 20 | Pig | PNG | 53 | 27 | 58 | 66 | 12 | 57 | 81 | 60 |
| GP 42 | Pig | PNG | 54 | 28 | 62 | 67 | 43 | 58 | 82 | 61 |
| GP6 | Pig | PNG | 55 | 28 | 62 | 68 | 43 | 58 | 82 | 61 |
| GP 28 | Pig | PNG | 56 | 28 | 62 | 69 | 43 | 58 | 83 | 61 |
| GP 14 | Pig | PNG | 57 | 29 | 62 | 70 | 43 | 58 | 82 | 62 |
| V1 H 106 | Human | PNG | 58 | 30 | 63 | 71 | 44 | 59 | 84 | 63 |
| V1 D 1 | Dog | PNG | 59 | 4 | 3 | 72 | 44 | 59 | 85 | 63 |
| V1 H 126 | Human | PNG | 60 | 27 | 64 | 73 | 45 | 59 | 86 | 63 |
| IMR 2 | Human | PNG | 61 | 31 | 65 | 74 | 46 | 60 | 87 | 64 |
| V1 H 117 | Human | PNG | 62 | 4 | 66 | 75 | 15 | 55 | 87 | 65 |
| H54 | Human | Australia | 63 | 32 | 67 | 76 | 15 | 61 | 88 | 6 |
| Edman | Human | Australia | 64 | 32 | 68 | 77 | 47 | 62 | 89 | 66 |
| IMR 81 | Human | PNG | 65 | 33 | 69 | 78 | 48 | 63 | 90 | 67 |
| IMR 48 | Human | PNG | 66 | 4 | 70 | 79 | 49 | 63 | 91 | 62 |
| IMR 49 | Human | PNG | 67 | 4 | 70 | 80 | 49 | 63 | 92 | 68 |
| WesB | Human | Australia | 68 | 5 | 71 | 39 | 34 | 64 | 93 | 69 |
| Wand G | Pig | Australia | 69 | 5 | 71 | 39 | 34 | 64 | 93 | 70 |
| 3295.90B | Pig | Australia | 70 | 5 | 71 | 39 | 34 | 64 | 93 | 71 |
| COF10 | Pig | Australia | 71 | 5 | 71 | 39 | 34 | 64 | 94 | 72 |
| Q98.0033.72 | Chicken | Australia | 72 | 5 | 71 | 39 | 34 | 64 | 95 | 72 |
| Q98.0072.08 | Chicken | Australia | 73 | 5 | 71 | 39 | 34 | 65 | 93 | 72 |
| Q98.0228.5.2 | Chicken | Australia | 73 | 5 | 71 | 39 | 34 | 65 | 93 | 72 |
| Wind Grower 6 | Pig | Australia | 74 | 34 | 71 | 39 | 50 | 54 | 96 | 73 |
| 42167 | Chicken | USA | 75 | 35 | 72 | 39 | 51 | 65 | 97 | 74 |
| UNL-3 | Pig | USA | 76 | 36 | 73 | 81 | 52 | 66 | 98 | 75 |
| P43/6/78^T^ | Pig | UK | 77 | 37 | 74 | 39 | 52 | 67 | 99 | 76 |
| GP 32 | Pig | PNG | 78 | 38 | 75 | 82 | 53 | 68 | 100 | 77 |
| GP 49 | Pig | PNG | 79 | 39 | 76 | 83 | 53 | 68 | 80 | 77 |
| GP 35 | Pig | PNG | 80 | 34 | 77 | 84 | 53 | 68 | 101 | 77 |
| GP 44 | Pig | PNG | 81 | 34 | 77 | 85 | 54 | 68 | 80 | 78 |
| 95/1000 | Pig | Australia | 82 | 40 | 78 | 86 | 55 | 69 | 93 | 79 |
| H60-2 | Human | Australia | 83 | 40 | 79 | 87 | 56 | 70 | 26 | 80 |
| UNL-5 | Pig | USA | 84 | 44 | 84 | 90 | 60 | 38 | 106 | 84 |
| Q97.000.6.8 | Chicken | Australia | 85 | 3 | 11 | 12 | 9 | 8 | 16 | 10 |
| Q97.000.6.4 | Chicken | Australia | 86 | 3 | 11 | 12 | 9 | 8 | 15 | 10 |
| Q97.000.6.2 | Chicken | Australia | 86 | 3 | 11 | 12 | 9 | 8 | 15 | 10 |
| Q97.000.6.7 | Chicken | Australia | 87 | 3 | 12 | 12 | 9 | 8 | 17 | 10 |
| V1 H 120 | Human | PNG | 88 | 3 | 13 | 13 | 9 | 9 | 18 | 11 |
| AN4170/01 | Pig | Sweden | 89 | 3 | 14 | 14 | 10 | 10 | 19 | 12 |
| 89/1069 | Pig | Canada | 90 | 3 | 15 | 15 | 11 | 11 | 20 | 13 |
| Margaret | Human | Australia | 91 | 3 | 16 | 16 | 11 | 12 | 14 | 14 |
| GP 5 | Pig | PNG | 92 | 3 | 17 | 17 | 12 | 13 | 21 | 15 |
| GP 3 | Pig | PNG | 93 | 3 | 18 | 18 | 13 | 13 | 22 | 16 |
| AN991/02 | Pig | Sweden | 94 | 3 | 19 | 19 | 14 | 14 | 23 | 99 |
| Wind W25 | Pig | Australia | 95 | 3 | 48 | 47 | 33 | 42 | 58 | 24 |
| Q1588.5 | Pig | Australia | 96 | 3 | 20 | 20 | 15 | 15 | 24 | 17 |
| ARD 127 | Pig | UK | 97 | 3 | 21 | 21 | 15 | 16 | 25 | 18 |
| Sonny | Human | Australia | 98 | 3 | 22 | 22 | 16 | 17 | 26 | 19 |
| HRM 4B | Human | Italy | 99 | 3 | 23 | 23 | 16 | 17 | 27 | 19 |
| Richelle | Human | Australia | 100 | 4 | 8 | 3 | 16 | 17 | 28 | 19 |
| Naomi | Human | Australia | 101 | 3 | 8 | 3 | 16 | 17 | 29 | 19 |
| Willow 7 | Pig | Australia | 102 | 11 | 24 | 24 | 17 | 18 | 30 | 20 |
| Willow IA8 | Pig | Australia | 103 | 3 | 24 | 25 | 18 | 19 | 31 | 21 |
| Q97.2110.4.1 | Chicken | Australia | 104 | 12 | 25 | 26 | 19 | 20 | 32 | 22 |
| Q97.000.6.22 | Chicken | Australia | 105 | 3 | 25 | 26 | 20 | 21 | 33 | 23 |
| Wand 015/C138 | Pig | Australia | 106 | 13 | 26 | 27 | 21 | 22 | 34 | 24 |
| 88-3769 | Pig | Canada | 107 | 14 | 27 | 28 | 21 | 23 | 35 | 25 |
| AN76/92 | Pig | Sweden | 108 | 3 | 28 | 29 | 21 | 24 | 36 | 26 |
| KeltonP5 | Pig | Australia | 109 | 15 | 29 | 30 | 22 | 25 | 37 | 27 |
| KeltonP1 | Pig | Australia | 110 | 15 | 29 | 30 | 22 | 26 | 38 | 27 |
| 9803 | Pig | Australia | 111 | 15 | 30 | 31 | 23 | 19 | 39 | 28 |
| AN497/93 | Pig | Sweden | 112 | 15 | 31 | 32 | 21 | 27 | 40 | 29 |
| B1555A | Pig | USA | 113 | 21 | 55 | 52 | 39 | 6 | 66 | 47 |
| Q98.0072.31 | Chicken | Australia | 114 | 22 | 55 | 53 | 19 | 47 | 67 | 49 |
| IMR 39 | Human | PNG | 115 | 43 | 82 | 88 | 59 | 73 | 104 | 82 |
| C62 | Pig | Sweden | 116 | 43 | 83 | 89 | 22 | 74 | 105 | 83 |
| 89-2005B | Pig | Canada | 117 | 45 | 85 | 91 | 61 | 75 | 107 | 85 |
| 89-2005A | Pig | Canada | 118 | 47 | 86 | 92 | 61 | 23 | 108 | 86 |
| D17 | Dog | Australia | 119 | 24 | 60 | 60 | 16 | 52 | 76 | 56 |
| Jeramiah | Human | Australia | 120 | 24 | 61 | 61 | 42 | 53 | 77 | 57 |
| Gap 51.2 | Human | Australia | 121 | 23 | 59 | 59 | 41 | 50 | 74 | 55 |
| Karlos | Human | Australia | 122 | 26 | 56 | 59 | 16 | 51 | 75 | 55 |
| Marsia | Human | Australia | 123 | 42 | 81 | 54 | 58 | 72 | 103 | 38 |
| 89-223A | Pig | Canada | 124 | 46 | 87 | 93 | 62 | 76 | 109 | 87 |
| PWS/B | Pig | UK | 125 | 48 | 88 | 94 | 62 | 16 | 110 | 88 |
| Meyers K9-12 | Dog | USA | 126 | 49 | 89 | 95 | 63 | 77 | 111 | 89 |
| 16242-94 | Dog | USA | 127 | 50 | 90 | 95 | 64 | 78 | 112 | 90 |

*PNG, Papua New Guinea; NZ, New Zealand
