# Supplementary material for: The pathogenic intestinal spirochaete Brachyspira pilosicoli forms a diverse recombinant species demonstrating some local clustering of related strains and potential for zoonotic spread
Source: Gut Pathog. 2013 Aug 16;5:24. doi: 10.1186/1757-4749-5-24 (PMC3751851; doi:10.1186/1757-4749-5-24)
Supplement: Additional file 2: Table S2 — Results of the Shimodaira-Hasegawa test for the seven loci. [file 1757-4749-5-24-S2.docx]

**Supplementary Table 2.** Results of the Shimodaira-Hasegawa test for the seven loci

| Shimodaira-Hasegawa test for *adh* | | | | |
| --- | --- | --- | --- | --- |
| Tree | Steps | Diff Steps | P Value | Significantly Worse? |
| *adh* | 237.0 | <------ best | | |
| *alp* | 375.0 | 138.0 | 0.000 | Yes |
| *est* | 465.0 | 228.0 | 0.000 | Yes |
| *gdh* | 419.0 | 182.0 | 0.000 | Yes |
| *glp* | 394.0 | 157.0 | 0.000 | Yes |
| *pgm* | 415.0 | 178.0 | 0.000 | Yes |
| *thi* | 425.0 | 188.0 | 0.000 | Yes |

| Shimodaira-Hasegawa test for *alp* | | | | |
| --- | --- | --- | --- | --- |
| Tree | Steps | Diff Steps | P Value | Significantly Worse? |
| *adh* | 1623.0 | 706.0 | 0.000 | Yes |
| *alp* | 917.0 | <------ best | | |
| *est* | 1656.0 | 739.0 | 0.000 | Yes |
| *gdh* | 1487.0 | 570.0 | 0.000 | Yes |
| *glp* | 1509.0 | 592.0 | 0.000 | Yes |
| *pgm* | 1542.0 | 625.0 | 0.000 | Yes |
| *thi* | 1556.0 | 639.0 | 0.000 | Yes |

| Shimodaira-Hasegawa test for *est* | | | | |
| --- | --- | --- | --- | --- |
| Tree | Steps | Diff Steps | P Value | Significantly Worse? |
| *adh* | 6403.0 | 4708.0 | 0.000 | Yes |
| *alp* | 5659.0 | 3964.0 | 0.000 | Yes |
| *est* | 1695.0 | <------ best | | |
| *gdh* | 6374.0 | 4679.0 | 0.000 | Yes |
| *glp* | 5958.0 | 4263.0 | 0.000 | Yes |
| *pgm* | 6206.0 | 4511.0 | 0.000 | Yes |
| *thi* | 5845.0 | 4150.0 | 0.000 | Yes |

| Shimodaira-Hasegawa test for *gdh* | | | | |
| --- | --- | --- | --- | --- |
| Tree | Steps | Diff Steps | P Value | Significantly Worse? |
| *adh* | 478.0 | 253.0 | 0.000 | Yes |
| *alp* | 407.0 | 182.0 | 0.000 | Yes |
| *est* | 566.0 | 341.0 | 0.000 | Yes |
| *gdh* | 225.0 | <------ best | | |
| *glp* | 418.0 | 193.0 | 0.000 | Yes |
| *pgm* | 418.0 | 193.0 | 0.000 | Yes |
| *thi* | 489.0 | 264.0 | 0.000 | Yes |

| Shimodaira-Hasegawa test for *glp* | | | | |
| --- | --- | --- | --- | --- |
| Tree | Steps | Diff Steps | P Value | Significantly Worse? |
| *adh* | 1227.0 | 698.0 | 0.000 | Yes |
| *alp* | 1038.0 | 509.0 | 0.000 | Yes |
| *est* | 1224.0 | 695.0 | 0.000 | Yes |
| *gdh* | 1147.0 | 618.0 | 0.000 | Yes |
| *glp* | 529.0 | <------ best | | |
| *pgm* | 1139.0 | 610.0 | 0.000 | Yes |
| *thi* | 1104.0 | 575.0 | 0.000 | Yes |

| Shimodaira-Hasegawa test for *pgm* | | | | |
| --- | --- | --- | --- | --- |
| Tree | Steps | Diff Steps | P Value | Significantly Worse? |
| *adh* | 1866.0 | 890.0 | 0.000 | Yes |
| *alp* | 1719.0 | 743.0 | 0.000 | Yes |
| *est* | 1859.0 | 883.0 | 0.000 | Yes |
| *gdh* | 1715.0 | 739.0 | 0.000 | Yes |
| *glp* | 1806.0 | 830.0 | 0.000 | Yes |
| *pgm* | 976.0 | <------ best | | |
| *thi* | 1758.0 | 782.0 | 0.000 | Yes |

| Shimodaira-Hasegawa test for *thi* | | | | |
| --- | --- | --- | --- | --- |
| Tree | Steps | Diff Steps | P Value | Significantly Worse? |
| *adh* | 7079.0 | 3765.0 | 0.000 | Yes |
| *alp* | 6737.0 | 3423.0 | 0.000 | Yes |
| *est* | 6507.0 | 3193.0 | 0.000 | Yes |
| *gdh* | 7106.0 | 3792.0 | 0.000 | Yes |
| *glp* | 6726.0 | 3412.0 | 0.000 | Yes |
| *pgm* | 6775.0 | 3461.0 | 0.000 | Yes |
| *thi* | 3314.0 | <------ best | | |
