# Supplementary material for: The pathogenic intestinal spirochaete Brachyspira pilosicoli forms a diverse recombinant species demonstrating some local clustering of related strains and potential for zoonotic spread
Source: Gut Pathog. 2013 Aug 16;5:24. doi: 10.1186/1757-4749-5-24 (PMC3751851; doi:10.1186/1757-4749-5-24)
Supplement: Additional file 3: Table S3 — Results of the Shimodaira-Hasegawa test on the four concatenated trees that showed the greatest difference with combinations of three loci. [file 1757-4749-5-24-S3.docx]

**Supplementary Table 3.** Results of the Shimodaira-Hasegawa test on the four concatenated trees that showed the greatest difference with combinations of three loci

| Shimodaira-Hasegawa test for *adh-alp-pgm* | | | | | | | | | |
| --- | --- | --- | --- | --- | --- | --- | --- | --- | --- |
| Tree | | Steps | Diff Steps | | P Value | | Significantly Worse? | | |
| *adh-alp-pgm* | | 2165.0 | <------ best | | | | | | |
| *alp-thi-est* | | 2798.0 | 633.0 | | 0.000 | | Yes | | |
| *glp-adh-thi* | | 2573.0 | 408.0 | | 0.000 | | Yes | | |
| *pgm-gdh-alp* | | 2563.0 | 398.0 | | 0.000 | | Yes | | |
|  | | | | | | | | |  |
| Shimodaira-Hasegawa test for *alp-thi-est* | | | | | | | | |  |
| Tree | Steps | | | Diff Steps | | P Value | | Significantly Worse? |  |
| *adh-alp-pgm* | 13162.0 | | | 4206.0 | | 0.000 | | Yes |  |
| *alp-thi-est* | 8956.0 | | | <------ best | | | | |  |
| *glp-adh-thi* | 12058.0 | | | 3102.0 | | 0.000 | | Yes |  |
| *pgm-gdh-alp* | 13267.0 | | | 4311.0 | | 0.000 | | Yes |  |
|  | | | | | | | | | |
| Shimodaira-Hasegawa test for *glp-adh-thi* | | | | | | | | | |
| Tree | | Steps | Diff Steps | | P Value | | Significantly Worse? | | |
| *adh-alp-pgm* | | 7188.0 | 1060.0 | | 0.000 | | Yes | | |
| *alp-thi-est* | | 7026.0 | 898.0 | | 0.000 | | Yes | | |
| *glp-adh-thi* | | 6128.0 | <------ best | | | | | | |
| *pgm-gdh-alp* | | 7735.0 | 1607.0 | | 0.000 | | Yes | | |
|  | | | | | | | | | |
| Shimodaira-Hasegawa test for *pgm-gdh-alp* | | | | | | | | | |
| Tree | | Steps | Diff Steps | | P Value | | Significantly Worse? | | |
| *adh-alp-pgm* | | 3169.0 | 496.0 | | 0.000 | | Yes | | |
| *alp-thi-est* | | 3489.0 | 816.0 | | 0.000 | | Yes | | |
| *glp-adh-thi* | | 3378.0 | 705.0 | | 0.000 | | Yes | | |
| *pgm-gdh-alp* | | 2673.0 | <------ best | | | | | | |
