# Supplementary figures and images for: The pathogenic intestinal spirochaete Brachyspira pilosicoli forms a diverse recombinant species demonstrating some local clustering of related strains and potential for zoonotic spread
Source: Gut Pathog. 2013 Aug 16;5:24. doi: 10.1186/1757-4749-5-24 (PMC3751851; doi:10.1186/1757-4749-5-24)

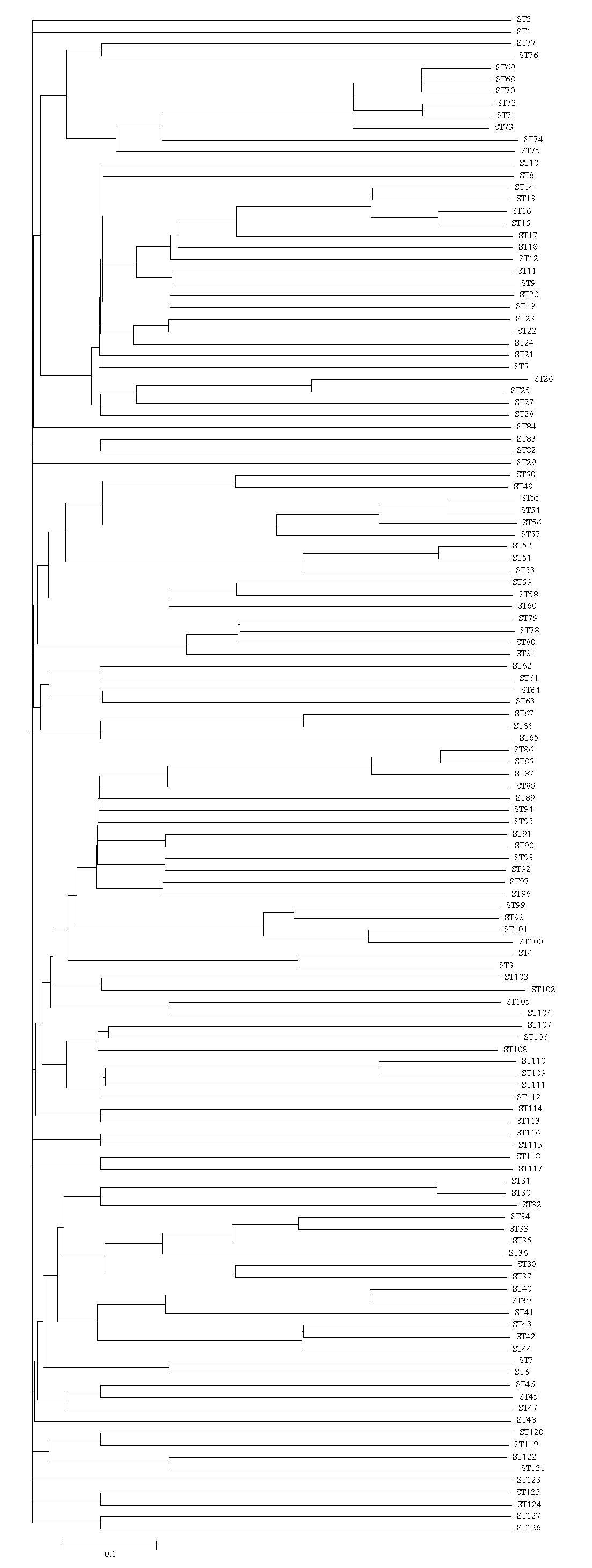

Supplement: Additional file 4: Figure S1 — Neighbour joining tree using the consensus sequences of the 131 B. pilosicoli isolates. A few localized clusters of isolates can be seen, with the largest being ST68 – ST73. [file 1757-4749-5-24-S4.tiff]
